# Mapping of lumbar multifidus stiffness Quantification in ankylosing spondylitis with shear-wave elastography

**DOI:** 10.1186/s12891-022-05854-0

**Published:** 2022-10-15

**Authors:** Mengyu Wang, Jia Liu, Lingcui Meng, Wen Fu, Jing Gao, Ruixia Ma, Yuxuan Luo, Yingjun Peng, Lihua Wu, Ziping Li

**Affiliations:** 1 School of Rehabilitation Medicine, Henan University of Chinese Medicine, 156 Jinshui East Rd, Zhengzhou, 450008 Henan Province China; 2 The Second Affiliated Hospital of Guangzhou University of Chinese Medicine, 111 Dade Road, Guangzhou, 510120 Guangdong Province China; 3 The First Affiliated Hospital of Henan University of Chinese Medicine, 19 Renmin Rd, Zhengzhou, 450099 Henan Province China; 4 The Second Clinical College of Guangzhou University of Chinese Medicine, 111 Dade Rd, Guangzhou, 510120 Guangdong Province China; 5 Huicheng Hospital of Traditional Chinese Medicine, 33 Maixing Rd, Huizhou, 516000 Guangdong Province China; 6 Shenzhen Bao’an Traditional Chinese Medicine Hospital Group, Guangzhou University of Chinese Medicine, 25 Yu’an 2nd Road, Shenzhen, 518000 Guangdong Province China

**Keywords:** Musculoskeletal ultrasound, Shear wave elastography, Ankylosing spondylitis

## Abstract

**Background:**

Lower back pain and stiffness are the typical symptoms of ankylosing spondylitis (AS). In this study, muscle mass was assessed by muscle density, mechanical elasticity, and area. We investigated the characteristics of lumbar paraspinal-muscle (PSM) mass using muscle ultrasound shear-wave elastography (SWE), as well as the validity of this method for identifying patients with AS.

**Methods:**

We recruited a representative cohort of 30 AS patients, and 27 healthy volunteers who were age- and sex-matched to the patient study group, investigated the Young’s modulus (YM), cross-sectional area (CSA) and thickness of lumbar multifidus (LM) muscle using SWE. This study did not need to be randomized. Data were collected at the department of ultrasonography of Guangdong Provincial Hospital of Chinese Medicine. We analyzed the data using SPSS version 18.0 (IBM Corp, Armonk, NY, USA). Normal distribution was evaluated by the Shapiro–Wilk test and Q–Q plots. Demographic and baseline data will be analyzed with standard descriptive statistics. Data will be presented as the mean ± standard deviation (SD). Non-normally distributed data are presented as medians with interquartile ranges (IQR).

**Results:**

Young’s modulus (YM) of SWE in AS patients was significantly higher than that in volunteers. Percentage change in lumbar multifidus (LM) muscle cross-sectional area (CSA) and thickness were significantly lower in AS patients than in healthy volunteers on the left side of the body. Correlation analysis showed a positive correlation between percentage change in CSA and thickness in both volunteers and AS patients. In AS patients, YM was negatively correlated with percentage change of CSA and thickness on the right side, while increased disease duration in AS was associated with increased YM on the left.

**Conclusion:**

AS patients showed reductions in LM muscle mass and function as the disease progressed, SWE could reflect these changes well.

Trial registration.

Chinese Clinical Trial Registry, ChiCTR2000031476. Registered 02/04/2020. http://www.chictr.org.cn/index.aspx.

## Background

Ankylosing spondylitis (AS) is a systemic disease characterized by chronic inflammation of the axial joint involving the sacroiliac joint (SIJ), with primary clinical symptoms of muscle stiffness, limited flexibility, and arthralgia [1]. Patients with AS suffer from chronic inflammation that attacks muscles and joints, leading to reduced muscle mass and joint damage. There are concerns that reduction in muscle mass might impair exercise tolerance and physical fitness and adversely affect emotional well-being [2]. Laboratory indicators can reflect disease inflammation levels, while computed tomography (CT) or magnetic resonance imaging (MRI) can assess the extent of bone damage. However, muscle stiffness caused by AS is often ignored, and there is no good, standard outcome measure for the assessment of this symptom.

Ultrasound (US) has become part of the fundamental Outcome Measures in Rheumatology (OMERACT) validation methodology, which uses repeated exercises to assess various domains, including inflammatory burden and structural damage [3, 4] US elastography is used to measure the degree of tissue distortion in response to an internal or external force. Elastography methods are divided into two types; strain elastography (SE) and shear wave elastography (SWE). SE is a semiquantitative technique that uses compression applied by the examiner to demonstrate tissue stifness. Although SE and SWE are equally effective in diagnosing some diseases, but SWE providing stiffness and shear wave velocity showed excellent performance at diagnosing diseases [5]. Compared with strain elastography, Shear-wave elastography (SWE) is based on so-called shear waves, which are induced within the tissue by a conventional US wave that interacts with the tissue, through which these horizontally directed waves propagate, and its low dependence on operator skills, because external compression or vibration is not needed [6]. The velocity of shear waves can be measured and processed to infer mechanical elasticity; in research systems, shear-wave attenuation and frequency dispersion are also used to calculate tissue viscosity [7]. Thus, SWE is more reproducible, objective, and quantitative than strain elastography. Since soft tissues such as tendons, ligaments, and joint capsules are viscoelastic in nature, their mechanical characteristics may be altered under applied strain, so we used SWE to avoid this problem [8, 9].

Muscle stiffness in this disease typically occurs in lumbar paraspinal-muscles (PSMs). Therefore, we devised a protocol using two-dimensional (2D) acoustic radiation to measure the longitudinal thickness and cross-sectional area (CSA) of lumbar PSMs (the levels of the L4–5 zygapophyseal joints using onscreen calipers) as a proxy for muscle force. To correlate lumbar PSM stiffness with muscle force. This highlights the potential of ultrasound SWE as a simple, real-time dynamic, and easily available alternative for the assessment of muscle stiffness in AS and as an extension of clinical rheumatic immune diseases and neuromuscular ultrasound for functional tests. The aim of this study was to show that SWE in a clinical US system with ultrafast algorithms to evaluate tissue composition[10, 11] and elasticity allowed the observation of muscle stiffness in AS.

## Methods

### Participants

The study was approved by the Institutional Ethics Committee of Guangdong Provincial Hospital of Chinese Medicine (Guangzhou, China; No. YF2019-232–01). From June 2020 to March 2021, patients and asymptomatic volunteers were prospectively included in our study. This part of non-invasive pilot study, which purpose was to investigate the characteristics of lumbar paraspinal-muscle (PSM) mass using ultrasound (US) shear-wave elastography (SWE), as well as the validity of this method for identifying patients with AS. This study did not need to be randomized. All subjects signed and provided the written informed consent before the experiment. Data were collected at the department of ultrasonography of Guangdong Provincial Hospital of Chinese Medicine.

Inclusion criteria for healthy volunteers were as follows: (1) no history of spine-related diseases; (2) no history of chronic lower back pain; (3) moderate body size (BMI ≤ 25), to avoid excessive observation error of the US probe; (4) 18–60 years of age; and (5) provision of written informed consent. Recruitment conditions for patients were as follows: (1) definite diagnosis of axial AS during a stable disease period; (2) 18–60 years of age, with onset age < 40 years; (3) X-ray imaging grade ≤ III; (4) course of disease ≤ 10 years; and (5) willingness to sign informed consent. Potential participants were excluded for the following reasons: (1) clinically important fracture of the spine; (2) spinal deformity or disability; (3) blood coagulation disorder; (4) presence of viral hepatitis, human immunodeficiency virus (HIV), or other blood infection; (5) pregnancy or lactation; (6) previous history of stroke or transient ischemic attacks; (7) pacemaker or other electrical device implanted; or (8) lack of consent, active pursuit of compensation or pending litigation.

### Ultrasound imaging

All volunteers and patients were scanned respectively by two sonographers in a prone position and a state of lumbar multifidus (LM) relaxation. Observer A had 10 years of experience in ultrasonic elastography, and observer B had 5 years of experience in ultrasonic elastography, the two observers specialize in musculoskeletal ultrasound. Each observer was blinded to the data of the other operator and the initial information of the enrolled subjects. The data from the first examination by observer A were used for further statistical analyses. We examined them using a Supersonic MACH 20 Imaging System with a linear-array transducer and a bandwidth of 4–15 MHz (Supersonic Imagine, Aix-en-Provence, France). All participants were tested separately on their left and right sides.

We measured elasticity, CSA, and LM thickness in the L4–5 horizontal core muscle group of the left and right lumbar spine. A pillow was placed under the subject’s pelvis to reduce lumbar lordosis for accurate measurement of the CSA area and LM thickness. We achieved a transverse plane by holding the transducer perpendicular to the long axis of the abdominal muscles. The lumbar muscle was measured twice: when the subject was relaxed in the prone position, and then again with the subject having one leg prone and the other raised back toward the torso to contract the core lumbar muscle. SWE provides semiquantitative (a color map) and quantitative (absolute SWE value) imaging biomarkers that are useful in assessing the elasticity of tendon and muscle composition and stiffness [12]. The map indicates the quality and reliability of the shear-wave measurements in a color-coded display (good = green, marginal = yellow, poor = red). We repeated the measurements until we achieved an optimal (green) map. Only minimal, constant contact force was applied with the transducer, as is generally recommended [13].

### Statistical analysis

We analyzed the data using SPSS version 18.0 (IBM Corp, Armonk, NY, USA). Normal distribution was evaluated by the Shapiro–Wilk test and Q–Q plots. We analyzed demographic and baseline data using standard descriptive statistics. For baseline data, we measured BMI using Mann–Whitney *U* test and sex using the chi-square test. Young’s modulus (YM) was assessed by the Mann–Whitney *U* test. Differences in values between resting and contracted LM muscle CSA and thickness, as well as percentage change in LM muscle CSA and thickness, were assessed using an independent-sample *t*-test. In AS, the relationship among mean YM, percent change in LM muscle thickness, and CSA were assessed using Spearman’s correlation coefficient (SCC). *P* < 0.05 was considered to indicate a statistically significant difference. To better quantify actual changes, we did not input missing values but used pairwise deletion, and all significance tests were two-tailed with α = 0.05.

## Results

Between June 2020 and March 2021, 30 AS patients and 29 volunteers who provided their informed consent were included. We excluded two volunteers, one with a family history of AS and the other with a history of chronic lower back pain. This left 30 AS patients and 27 volunteers for data analysis. The baseline demographic and clinical characteristics were showed in Table 1.

**Table 1:** Baseline characteristics in patients and volunteers

Variables	Patients (*n* = 30)	Controls (*n* = 27)	*P*
Sex (male/female)	27/3	23/4	0.697
BMI (kg/m^2^)	22.04 (19.12–25.89)	21.67 (20.45–23.38)	0.949
Left YM (kPa)	27.80 (16.10–43.33)	16.40 (11.40–22.50)	0.004
Right YM (kPa)	22.25 (15.73–38.08)	13.00 (9.90–19.20)	0.002
Left P-CSA (%)	11.12 (2.86–27.88)	28.89 (10.32–38.40)	0.020
Right P-CSA (%)	16.33 (2.11–29.75)	23.73 (19.14–34.98)	0.053
Left P- thickness (%)	9.44 (4.34–20.04)	21.50 (14.36–30.41)	0.001
Right P- thickness (%)	16.60 ± 10.71	20.39 ± 10.04	0.176 (95%CI: -9.311–1.743)

**Fig. 1:**
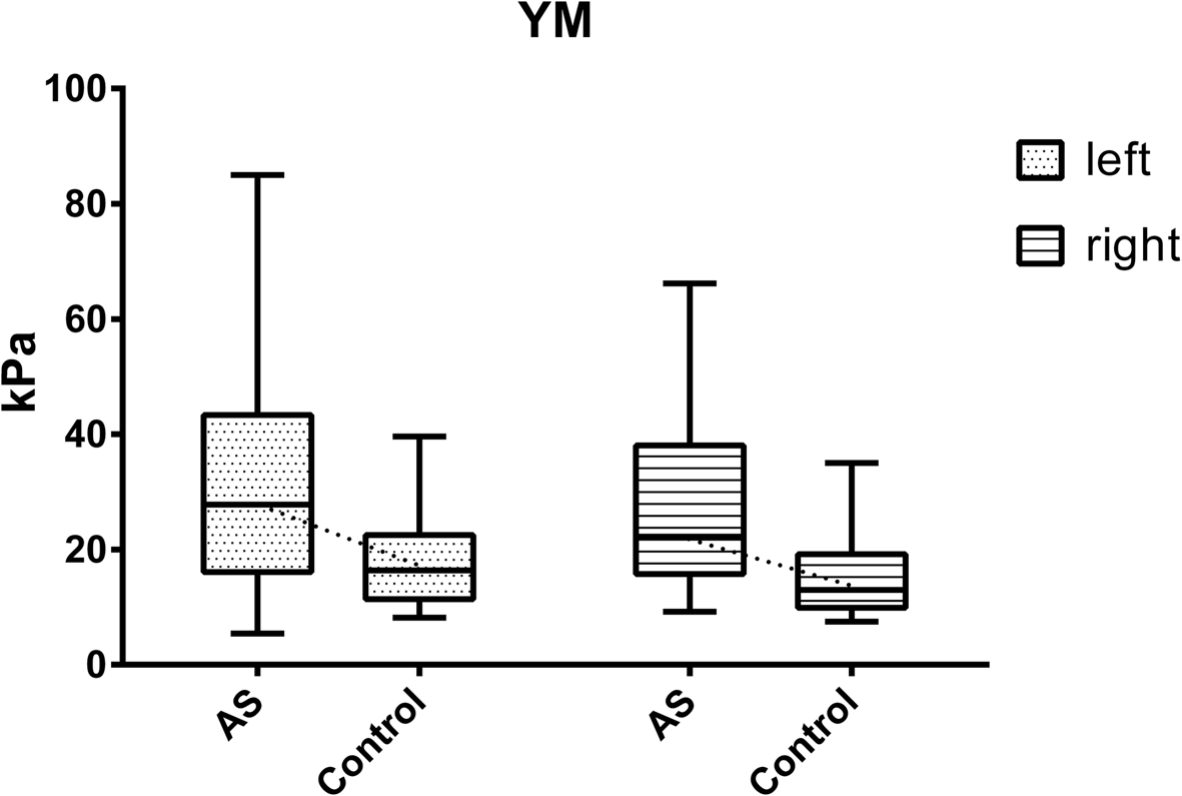
Box plots used to compare Young’ s modulus of the lumbar multifidus muscle on the left and right sides in patients and controls. Numbers are YM in kPa. The left and right LM muscles of the two groups were compared separately (left = blue, right = red). The ends of the boxes represent the upper and lower quartiles, and the line separating the two areas indicates the median. Vertical lines indicate the full ranges of values in the data

**Fig. 2:**
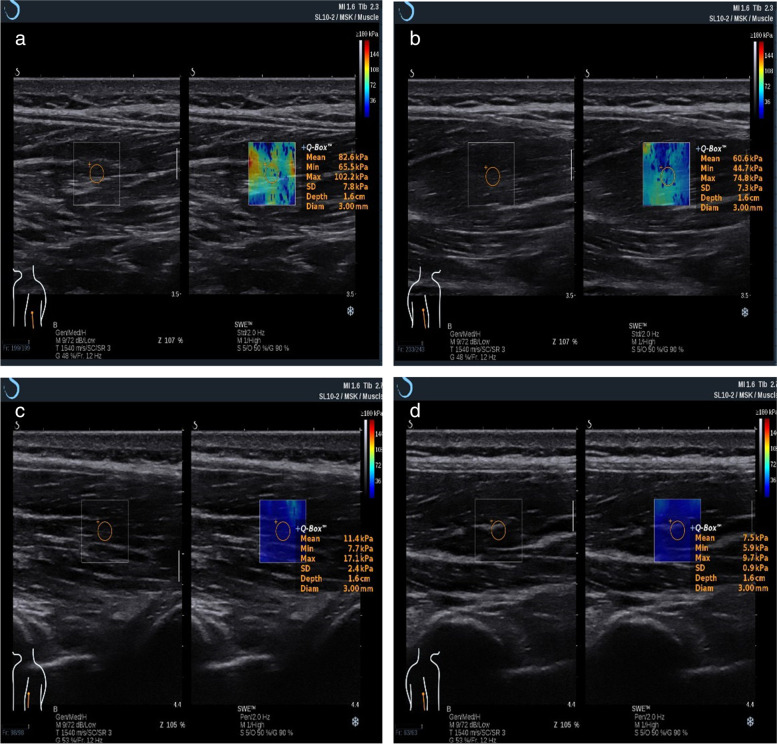
Representative images of elasticity measurement by shear-wave elastography (SWE) in one AS patient and one volunteer. **a** SWE US image of left LM muscle in AS patients. **b** SWE US image of right LM muscle in AS patients. **c** SWE US image of left LM muscle in controls. **d** SWE US image of right LM muscle in controls. The regions of interest (ROIs) were positioned at the level of the L4–5 zygapophyseal joints of the lumbar PSMs in the B-mode image. The lower and upper rows show changes in the ROIs by grayscale US and SWE, respectively. The color map shows the distribution of elasticity values scaled from 0 to 180 kPa and calculated from shear-wave velocity (SWV) values using YM

We analyzed 30 AS patients (ages, 29.53 ± 3.98 years; 3 women, 27 men) and 27 volunteers (ages, 27.48 ± 1.81 years; 4 women, 23 men). Figure 1 illustrates that the YM of LM in AS patients was significantly higher than in healthy controls on both the left and right sides (left: 27.80 [16.10–43.33] *vs.* 16.40 [11.40–22.50] kPa; *Z* = –2.901, *P* = 0.004; right: 22.25 [15.73–38.08] *vs.* 13.00 [9.90–19.20] kPa; *Z* = –3.053, *P* = 0.002). Compared with healthy volunteers, the quality of the LM muscle in AS patients was poor (Figs. 1 and 2).

**Fig. 3:**
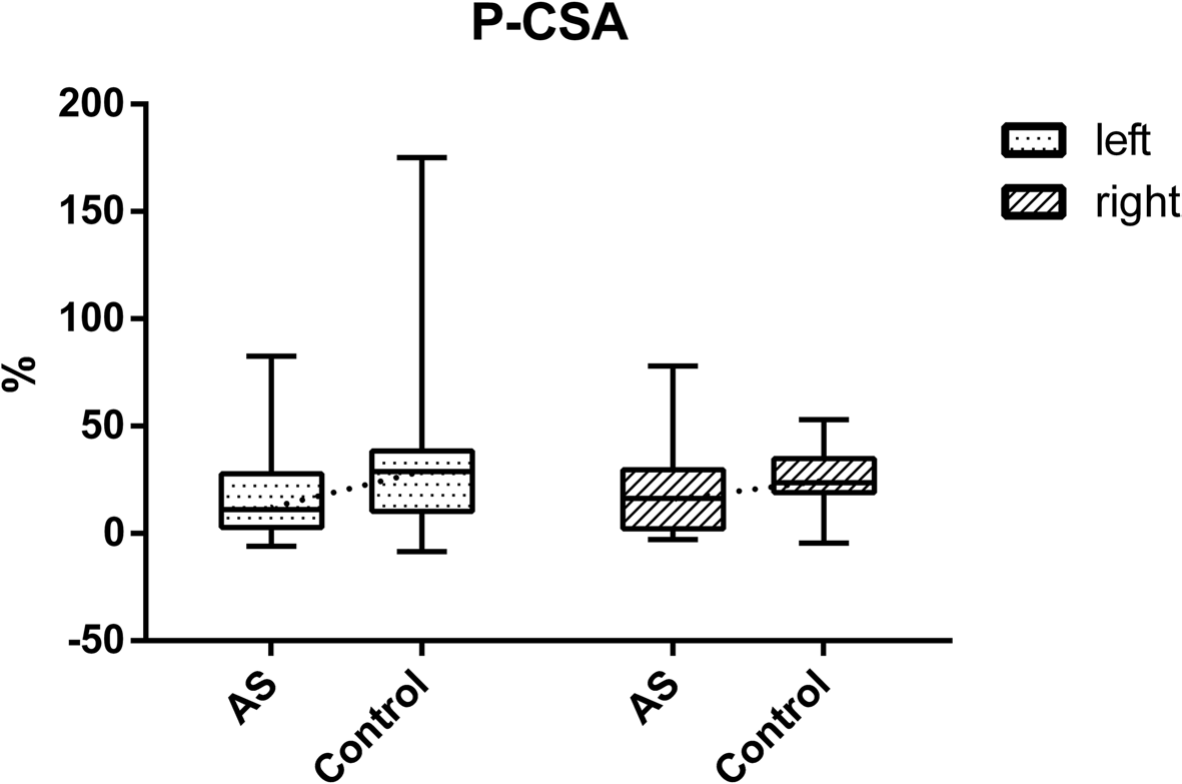
Box plots used to compare the percentage change in LM muscle CSA on the left and right. Numbers are CSA percentages. The ends of the boxes represent the upper and lower quartiles, and the line separating the two areas indicates the median. Vertical lines indicate the full ranges of values in the data

Percentage change in LM muscle CSA and thickness were calculated as contracted – rest/rest. Percent CSA change was significantly lower (Wilcoxon rank-sum test) in AS patients than in healthy volunteers on the left side, but there was no significant difference on the right side (left: 11.12 [2.86–27.88] *vs.* 28.89 [10.32–38.40] %; *Z* = –2.333, *P* = 0.020; right: 16.33 [2.11–29.75] *vs.* 23.73 [19.14–34.98] %; *Z* = –1.934, *P* = 0.053; Fig. 3).

**Fig. 4:**
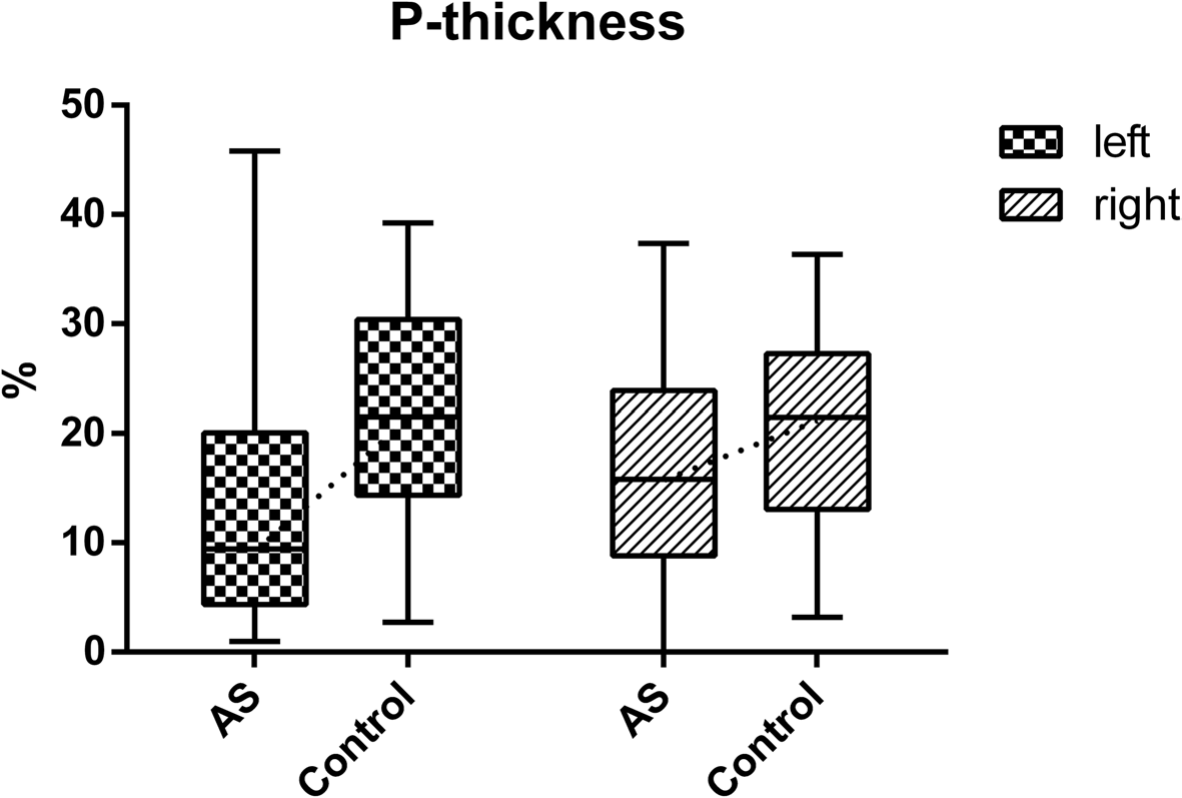
Box plots used to compare the percentage change in left LM muscle thickness on the left and right sides. The ends of the boxes indicate the upper and lower quartiles, and the line separating the two areas indicates the median. Vertical lines indicate the full ranges of values in the data. Symbol plots used to compare the percentage change in right LM muscle thickness. The ends of the symbol are the upper and lower standard deviation, and the dot in the middle indicates the mean. Numbers are thickness in percent signs

The left-side percentage change in LM muscle thickness was significantly lower in AS patients (Wilcoxon rank-sum test) than in healthy controls (9.44 [4.34–20.04] *vs.* 21.50 [14.36–30.41]; *Z* = –3.308, *P* = 0.001), but there was no significant difference (independent-sample *t* test) on the right side (16.60 ± 10.71 *vs.* 20.39 ± 10.04; *t* = –1.372, *P* = 0.176; Fig. 4).

**Fig. 5:**
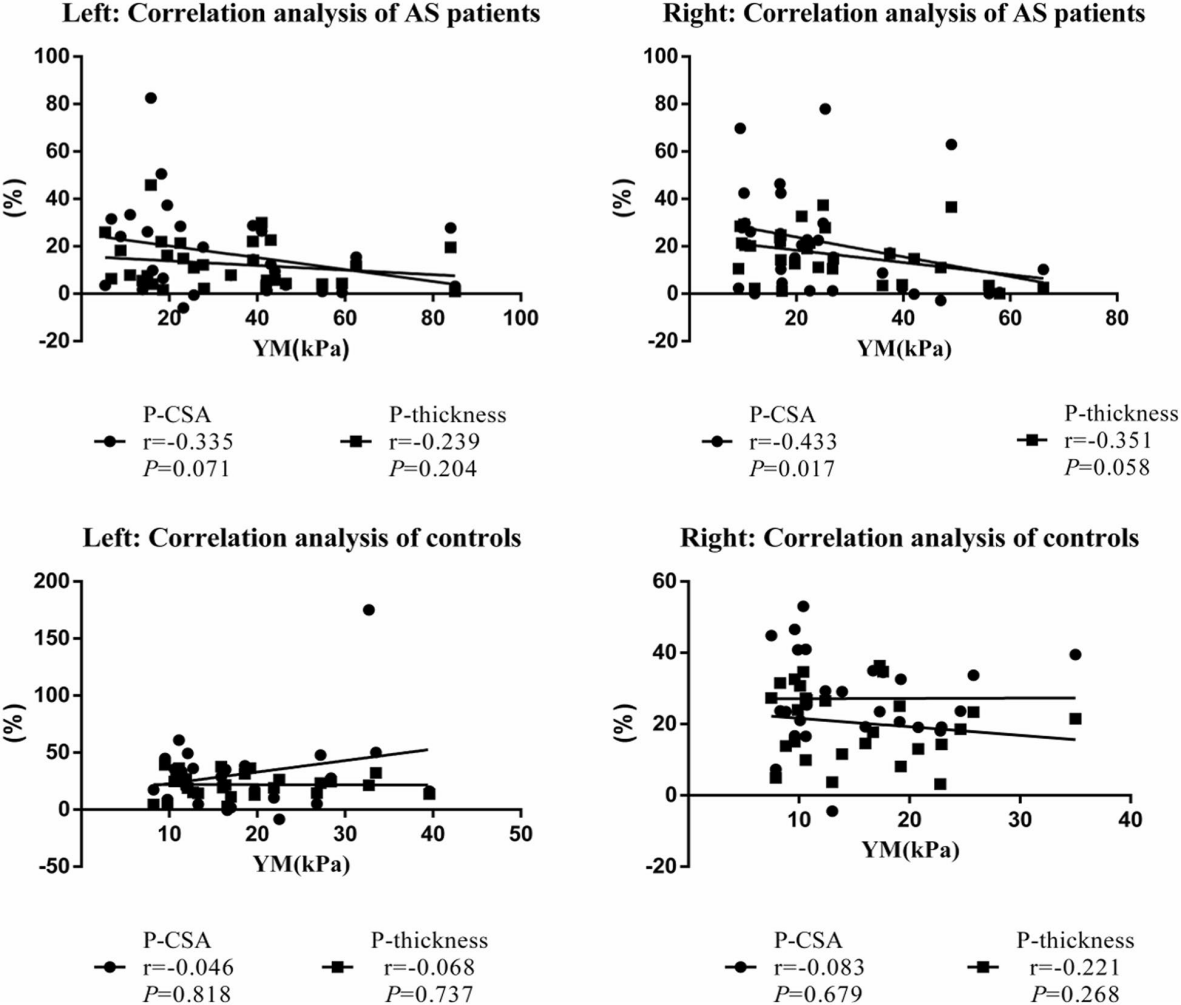
Scatter plot of the percentage change in thickness (P-thickness), percentage change in CSA (P- CSA), and YM in left and right LM in patients an AS. Trend line emphasizing linear correlation is depicted

**Fig. 6:**
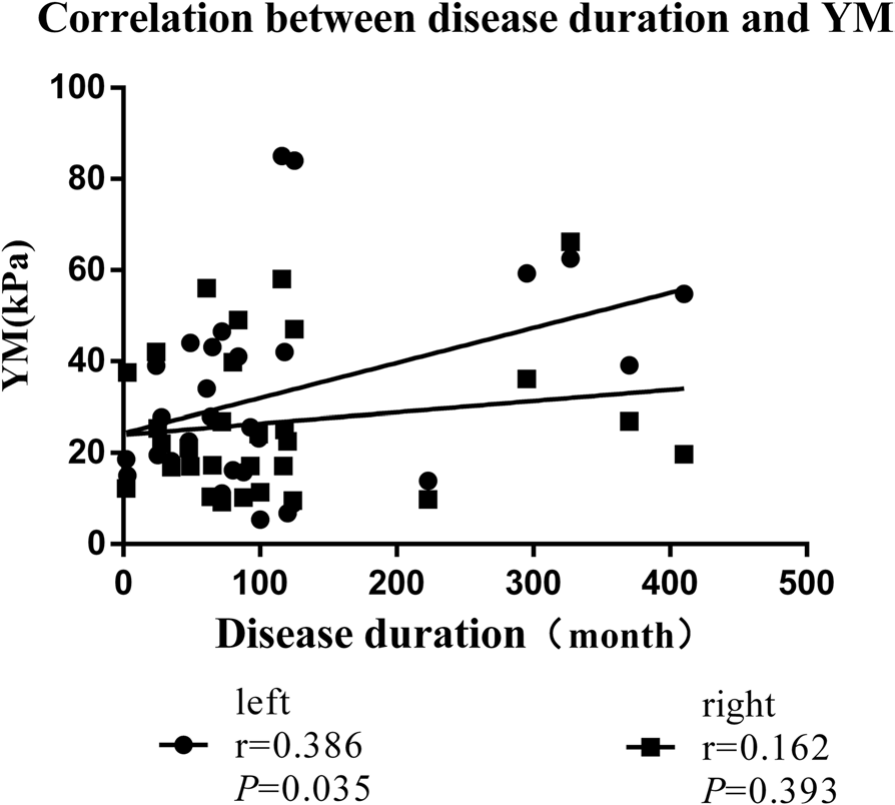
Scatter plot showing the relationship between percentage change of YM and disease duration on the left and right sides of the LM in AS patients. The trend line emphasizes linear correlation

Correlation analysis (nonparametric SCC; Fig. 5) showed a positive correlation between percentage change of CSA and thickness in controls (left: *r* = 0.592, *P* = 0.001; right: *r* = 0.627, *P* = 0.000) and AS patients (left: *r* = 0.566, *P* = 0.001; right: *r* = 0.785, *P* = 0.000). In AS patients, YM was negatively correlated with percentage change of CSA (*r* = –0.433, *P* = 0.017) and thickness (*r* = –0.351, *P* = 0.058) on the right of the LM, but we found no significant correlation on the left (CSA: *r* = –0.335, *P* = 0.071; thickness: *r* = –0.239, *P* = 0.204); there was also no correlation on either side in healthy controls (left: CSA: *r* = –0.046, *P* = 0.818; thickness: *r* = –0.068, *P* = 0.737; right: CSA: *r* = –0.083, *P* = 0.679; thickness: *r* = –0.221, *P* = 0.268; Fig. 5). Increased duration of AS was associated with increased YM (left: *r* = 0.386, *P* = 0.035; right: *r* = 0.162, *P* = 0.393; Fig. 6). We observed no significant correlation between disease duration and percentage change of CSA (left: *r* = –0.275, *P* = 0.141; right: *r* = –0.123, *P* = 0.517) or thickness (left: *r* = –0.168, *P* = 0.374; right: *r* = –0.204, *P* = 0.281).

**Fig. 7:**
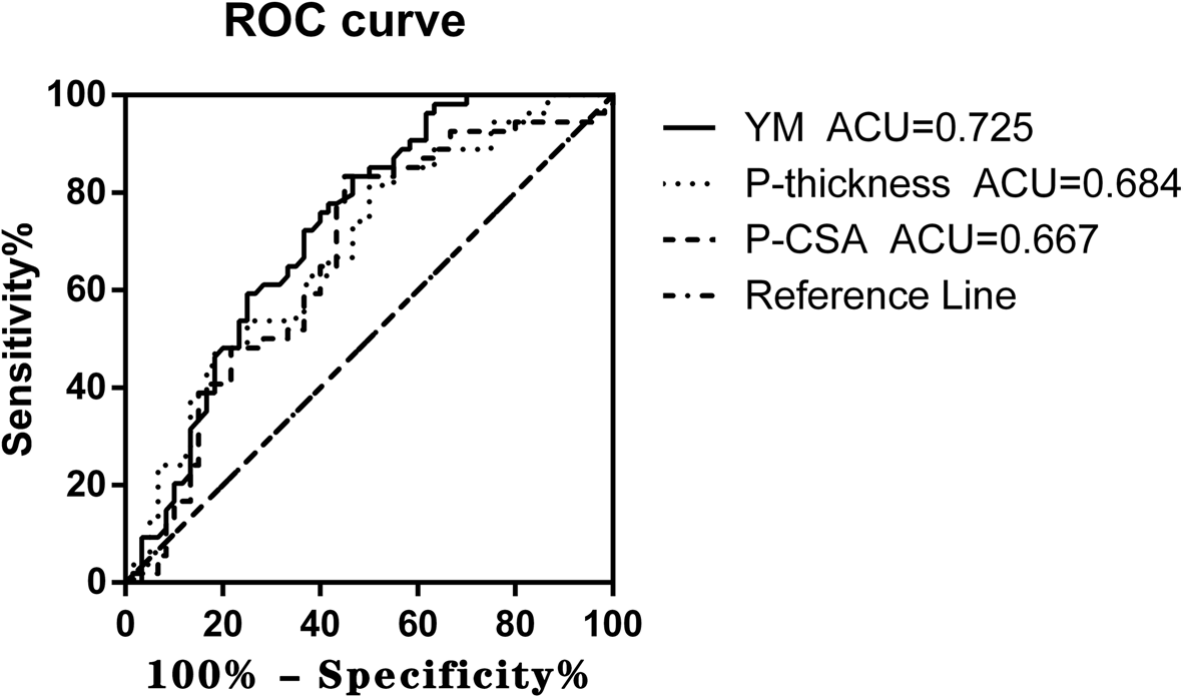
ROC analysis with AUC for each Ultrasound parameter difference used to identify AS patients. [AUC for SWE, P-CSA, P-thickness]

The area under the receiver operating characteristic (ROC) curve (AUC) for YM values for identification of AS in all participants is shown in Fig. 7. The evaluation of LM mass in AS patients had favorable validity for differences in YM values (AUC = 0.725 [0.632–0.818]; *P* < 0.0001), P-CSA difference values (AUC = 0.667 [0.566–0.768]; *P* = 0.0021) and P-thickness difference values (AUC = 0.684 [0.586–0.781]; *P* = 0.0007). The Youden index of YM was 0.366 (sensitivity, 83.33%; specificity, 53.33%), and the optimal cutoff value difference was 23.05 kPa. The P-CSA Youden index was 0.383 (sensitivity, 83.33%; specificity, 55%), and the optimal cutoff value difference was 15.78%. The P-thickness Youden index was 0.315 (sensitivity, 81.48%; specificity, 50%), and the optimal cutoff value difference was 12.75%.

**Fig. 8:**
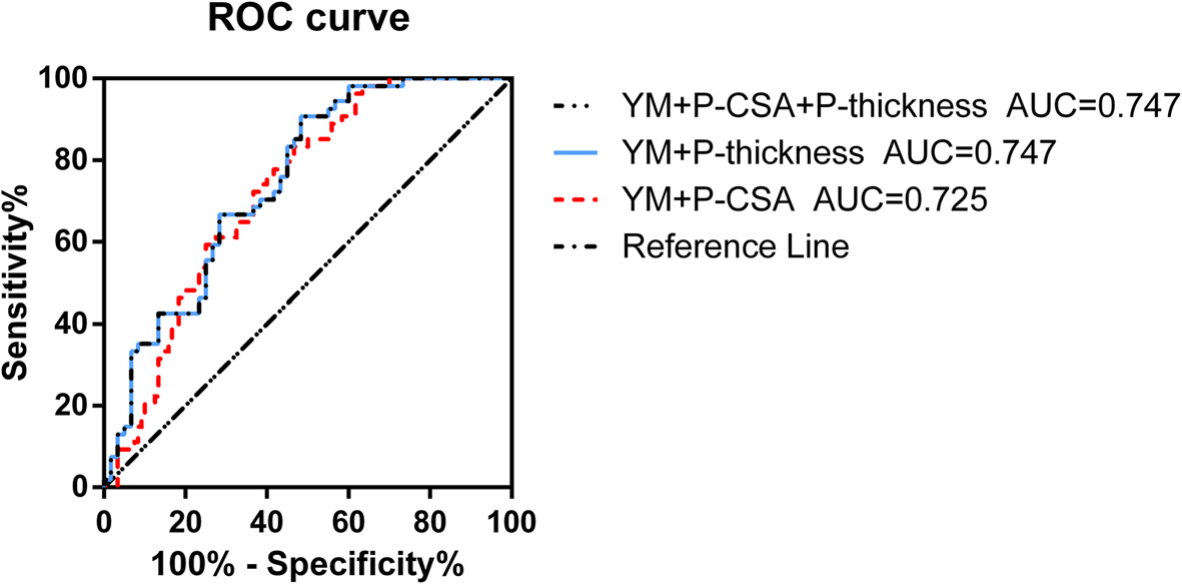
ROC analysis with AUC for combinations of Ultrasound parameters in differentiating AS patients. AUC, area under the curve; ROC, receiver operating characteristic

In our study, although using YM on its own offers better sonographic parameters in terms of differentiating AS patients. A combination of multiple parameters may improve diagnostic accuracy. Our study shows that YM + P-CSA + P-thickness (AUC = 0.747 [0.6580–0.8358]; *P* < 0.0001) and YM + P-thickness (AUC = 0.747 [0.6580 to 0.8358]; *P* < 0.0001) have higher diagnostic accuracy than YM + P-CSA (AUC = 0.725 [0.6319–0.8178]; *P* < 0.0001) (Fig. 8). The Youden index of YM + P-CSA + P-thickness was 0.424 (sensitivity, 90.74%; specificity, 51.67%), YM + P-thickness was 0.424 (sensitivity, 90.74%; specificity, 51.67%), YM + P-CSA was 0.366 (sensitivity, 83.33%; specificity, 53.33%). Results of our work confirmed that the additional diagnostic information provided by YM and P-thickness can be combined for yield improved diagnostic performance.

## Discussion

Ankylosing spondylitis is considered a so-called spondyloarthritis (SpA) disease, which refers to a group of immune-mediated diseases characterized by inflammation of the axial skeleton, peripheral joints, and entheses accompanied by muscle stiffness, causing disability [14]. Joint inflammation can be well measured by imaging and laboratory tests. However, while the symptom of muscle stiffness is easily ignored or rarely evaluated, it is also a cause of disability [15]. To date, few studies have compared PSM morphology and histology of AS patients with those of healthy controls. Quantitative, objective, and non-invasive methods for evaluating muscle stiffness of AS severity are needed to aid in diagnosis, advance the understanding of the natural history and pathophysiology of AS, and monitor therapeutic effects in clinical settings or controlled trials. Thus, we designed a pilot study to prove that ultrasound shear-wave elastography could objectively quantify LM muscle mass and function. We determined muscle stiffness as measured by SWE to be significantly higher in patients than in healthy volunteers. In addition, percentage changes in LM muscle CSA and thickness were significantly lower in AS patients than in healthy controls.

### Relationship between muscle stiffness and ankylosing spondylitis

Mechanical property is an essential factor for maintaining muscle function [16]. Meanwhile, decreased muscle stiffness can reduce muscle strength and power [17]. Loss of skeletal muscle mass is an objective measure of frailty that is associated with functional impairment and disability [18]. Quantifying muscle stiffness can help understand the mechanisms of these musculoskeletal symptoms and pathologies. In vivo biomechanical assessments provide non-invasive estimates of spinal stiffness that, together with other objective tests and outcome measures, can help clinicians distinguish spinal disorders from one another and treat affected patients [19]. In AS patients, increased disease duration is associated with decreased tissue elasticity or myofascial degradation [20], which is consistent with our findings. Patients with AS have more fatty degeneration and denervation in PSMs than non-radiographic axial SpA (nr-axSpA) patients or volunteers, and more matter at the L4–L5 and L5–S1 disc levels [21]. Loss of muscle CSA compatible with atrophy is present between levels L1 and L5 of the PSMs in AS, which is thought to be the result of various cytokines and limitations in spinal mobility [22]. This is consistent with our observations of LM muscle atrophy and increased stiffness. These processes might also contribute to the severity of pain and disability.

Considerable evidence indicates that US imaging has good to high intrarater reliability for assessing the LM muscles [23, 24]. In our study, two dependent variables represented muscle function: percent change in thickness and percentage change in CSA. SWE measurements reflect muscle strength and mass. Percent thickness change has been proven adequately reliable because clinical use of US imaging usually involves benign patient management decisions involving exercises for lumbar stabilization [25]. LM muscle CSA was significantly and positively correlated with LM muscle thickness at rest and during contraction [26].

### Diagnostic value of shear-wave elastography in ankylosing spondylitis

The diagnosis of AS is mainly based on symptoms, disease course, and imaging tests; questionnaires and laboratory examination are commonly used in follow-up assessments. However, questionnaires pose the risk of underestimation or overestimation. C-reactive protein (CRP) and erythrocyte sedimentation rate (ESR) are two acute-phase reactants that classically have been used to assess the presence of inflammation in patients. Unfortunately, they are nonspecific and of low sensitivity [27], especially in patients with nr-axSpA, where acute-phase proteins remain within normal limits most of the time [28]. Therefore, objective indicators of symptoms are important for early diagnosis, surveillance, and evaluation of therapeutic efficacy. In this study, ROC curve analysis showed that differences in phase values were valid for identifying patients with AS. Our results showed that the optimal cutoff value for the LM (23.05) SWE had clinical diagnostic value for early identification of AS patients (sensitivity, 83.33%). Importantly, because the stiffness of the lower back is not specific to AS, specificity was just 53.33%. YM combined with P-thickness can improve the sensitivity (90.74%) of AS disease identification.

### Study limitations

There were several limitations to this study. First, large sample sizes were needed. Second, this was an observational study without other support from imaging or neuroendocrine blood results. Third, technical issues and lack of standardization posed limitations on SWE use in the assessment of tendon injuries.

## Conclusion

In both AS patients and healthy subjects, correlations between percentage change of CSA and percentage change of thickness were similar, suggesting that the disease did not directly alter their inherent interrelations. By contrast, decreased lumbar-muscle elasticity (the inverse of decrement) was primarily correlated with the percentage change in CSA and thickness in subjects with AS. Whereas, the combined diagnosis of YM and P-thickness seems to be more meaningful for reflecting muscle function and identifying AS diseases. The novel result was that disease and duration differentially affected stiffness and elasticity respectively; this deserves further investigation into the relevant biomechanical properties and their underlying mechanisms. Our results could help guide the development of a specific exercise program to rehabilitate PSMs in the early stages of AS, which might help clinicians better assess balance problems, disability, pain, and degenerative processes of the spine in these patients.

## Data Availability

The datasets used and analysed during the current study are available from the corresponding author on reasonable request. The data related to this study can be made available upon reasonable request.

## Abbreviations

AS: Ankylosing spondylitis;
PSM: Paraspinal-muscle
SWE: Shear-wave elastography
YM: Young’s modulus
LM: Lumbar multifidus
CSA: Cross-sectional area
SIJ: Sacroiliac joint
CT: Computed tomography
MRI: Magnetic resonance imaging
US: Ultrasound
OMERACT: Outcome Measures in Rheumatology
2D: Two-dimensional
ROIs: Regions of interest
SWV: Shear-wave velocity
ROC: Receiver operating characteristic
SpA: Spondyloarthritis
nr-axSpA: Non-radiographic axial SpA
CRP: C-reactive protein
ESR: Erythrocyte sedimentation rate
